# European survey on preanalytical sample handling – Part 1: How do European laboratories monitor the preanalytical phase? On behalf of the European Federation of Clinical Chemistry and Laboratory Medicine (EFLM) Working Group for the Preanalytical Phase (WG-PRE)

**DOI:** 10.11613/BM.2019.020704

**Published:** 2019-06-15

**Authors:** Janne Cadamuro, Giuseppe Lippi, Alexander von Meyer, Mercedes Ibarz, Edmee van Dongen, Lases, Michael Cornes, Mads Nybo, Pieter Vermeersch, Kjell Grankvist, Joao Tiago Guimaraes, Gunn B.B. Kristensen, Barbara de la Salle, Ana-Maria Simundic

**Affiliations:** 1 Department of Laboratory Medicine, Paracelsus Medical University, Salzburg, Austria; 2 Section of Clinical Chemistry, University of Verona, Verona, Italy; 3 Institute of Laboratory Medicine, Kliniken Nordoberpfalz AG and Klinikum St. Marien, Weiden and Amberg, Germany; 4 Department of Laboratory Medicine, University Hospital Arnau de Vilanova, IRBLleida, Lleida, Spain; 5 Department of Clinical Chemistry, Amsterdam UMC, University of Amsterdam, Amsterdam, The Netherlands; 6 Clinical Chemistry Department, Worcestershire Acute Hospitals NHS Trust, Worcester, UK; 7 Department of Clinical Biochemistry and Pharmacology, Odense University Hospital, Odense, Denmark; 8 Clinical Department of Laboratory Medicine, University Hospitals Leuven, Leuven, Belgium; 9 Department of Medical Biosciences, Clinical Chemistry, Umea University, Umea, Sweden; 10 Department of Clinical Pathology, São João Hospital Center, Department of Biomedicine, Faculty of Medicine, and EPI Unit, Institute of Public Health, University of Porto, Porto, Portugal; 11 Norwegian Quality Improvement of laboratory examinations (Noklus), Bergen, Norway; 12 UK NEQAS Haematology, West Hertfordshire Hospitals NHS Trust, operating UK NEQAS for Haematology and Transfusion, Watford, UK; 13 Department of Medical Laboratory Diagnostics, University Hospital Sveti Duh, Zagreb, Croatia

**Keywords:** preanalytics, standardization, survey

## Abstract

**Introduction:**

Compared to other activities of the testing process, the preanalytical phase is plagued by a lower degree of standardization, which makes it more vulnerable to errors. With the aim of providing guidelines and recommendations, the EFLM WG-PRE issued a survey across European medical laboratories, to gather information on local preanalytical practices. This is part one of two coherent articles, which covers all practices on monitoring preanalytical quality except haemolysis, icterus and lipemia (HIL).

**Materials and methods:**

An online survey, containing 39 questions dealing with a broad spectrum of preanalytical issues, was disseminated to EFLM member countries. The survey included questions on willingness of laboratories to engage in preanalytical issues.

**Results:**

Overall, 1405 valid responses were received from 37 countries. 1265 (94%) responders declared to monitor preanalytical errors. Assessment, documentation and further use of this information varied widely among respondents and partially among countries. Many responders were interested in a preanalytical online platform, holding information on various aspects of the preanalytical phase (N = 1177; 87%), in a guideline for measurement and evaluation of preanalytical variables (N = 1235; 92%), and in preanalytical e-learning programs or webinars (N = 1125; 84%). Fewer responders were interested in, or already participating in, preanalytical EQA programs (N = 951; 71%).

**Conclusion:**

Although substantial heterogeneity was found across European laboratories on preanalytical phase monitoring, the interest in preanalytical issues was high. A large majority of participants indicated an interest in new guidelines regarding preanalytical variables and learning activities. This important data will be used by the WG-PRE for providing recommendations on the most critical issues.

## Introduction

The preanalytical phase of laboratory testing has long been known as the most vulnerable part of the total testing process, potentially leading to misidentifications, transportation/storage errors or erroneous results due to poor sample quality (haemolysis) or contamination, which can ultimately lead to patient harm (*1*, *2*). This mostly arises from many small risks eventually merging into an exponential risk of failure. More specifically, the fact that preanalytical processes predominantly take place outside the laboratory, makes it challenging to establish and enforce quality control measures comparable to those used as standard throughout the analytical processes. They also involve many different stakeholders such as patients, clinicians, nurses and logistics personnel, thus the potential variables contributing to the deviation of test results are numerous (*3*). Despite these acknowledged facts, which influence the analytical outcome and potentially impair both patient diagnosis and management, relatively minor initiatives have been made to overcome this problem compared to the analytical phase. Preanalytical errors are widely monitored, but this practice is often limited to detecting haemolysis rates in a subset of samples received by the laboratory and noting the number of detected preanalytical errors related to received test tubes and test requests. These investigations were again performed in a very heterogeneous way, thus making it hard to perform reliable benchmarking (*4*-*7*). The reasons may include the lack of guidelines for health care staff to follow. However, even if guidance is available on a specific preanalytical process, adherence may often be limited as Simundic *et al.* demonstrated for venous blood collection (*8*).

In the last decade, preanalytical issues have been addressed more extensively, with dedicated working groups being developed throughout Europe. In 2012, the European Federation of Clinical Chemistry and Laboratory Medicine (EFLM) established a Working Group for “Preanalytical Phase” (WG-PRE). It began with five core members, evolving into a large and productive group, currently resembling members from 17 European countries. The terms of reference for this WG include the provision of operating procedures and consensus guidelines based on current evidence in order to standardize and harmonize preanalytical processes, thus minimizing the risk of errors.

The potential areas in the preanalytical process requiring optimization/standardization emerge from all issues related to phlebotomy, transport or handling of preanalytically altered samples, among others. In order to identify the areas for which guidance is most urgently needed, a status of current practices on preanalytical topics is needed. Therefore, the aim of this study was to survey European countries on questions regarding monitoring, documentation and evaluation of preanalytical errors, handling of haemolytic, lipemic or icteric samples, communication with clinicians and nurses about preanalytical topics, and the willingness/interest of laboratory professionals to focus more on these issues.

Due to the large amount of data, the results and insights from this survey were split into two coherent manuscripts. This first article presents the survey and subsequently displays and discusses if, and how, laboratories across Europe monitor the preanalytical phase and how data from this monitoring is currently used. Additionally, it focusses on the interest of participants in preanalytical issues and their willingness to actively engage in this topic. The second article presents and discusses the results of questions on haemolysis, lipemia and icterus monitoring (*9**)*).

## Materials and methods

A survey was developed by the WG-PRE, containing 39 questions, which covered general demographic information as well as specific aspects regarding the identification of preanalytical issues, haemolysis, icterus and lipemia, the evaluation of preanalytical measurements and the improvement of respective processes using their outcomes along with standardization of preanalytical issues. All questions and respective answering options are shown in Supplemental Table 1.

To deliver the survey, an electronic online survey tool was initially used (SurveyMonkey, San Mateo, USA). Before widespread dissemination across European laboratories, the survey was piloted in Austria in 2016. Based on the results, the survey was reconfigured, and an alternate survey tool was finally used, which better fitted the aims of this project (LimeSurvey, LimeSurvey GmbH, Hamburg, Germany). Some questions were shown to participants based on their answers to previous questions.

After approval by the EFLM Scientific Committee and the EFLM Executive Board, the survey was sent through the European Organisation for External Quality Assurance Providers in Laboratory Medicine (EQALM) network or EFLM national societies (when an EQALM organization was unavailable in the country) to members, with a request to further distribute the survey link, accompanied by an invitation letter to participating laboratories. Participants were asked to only fill in the online form once *per* laboratory between October 1^st^ and November 30^th^, 2017.

Evaluation of results was performed using IBM SPSS Statistics V.24 (IBM, Armonk, New York, USA). Answers from non-EFLM member countries were not incorporated in this evaluation. In country-specific sub-analyses, countries with only five respondents or less were also eliminated since these answers may not reflect the situation for the entire country. Content analysis on differences and similarities within the text was used to analyse the written responses to the open-ended question: ”Which preanalytical topics concern you the most?“ (*10*). According to the journals guideline, percentages are rounded and shown in whole numbers, except those < 10% if necessary and applicable (*11*).

## Results

Overall, 1416 participants from 45 countries completed the survey. Eleven of these responses were removed as they were provided by non-EFLM member countries, leaving 1405 responses from 37 countries. Another 58 responders stated that they were not involved in analysing blood samples. These participants were not introduced to the remaining questions. A list of the remaining 1347 participants including the number of responses is shown in Table 1. A representative number of primary care laboratories as well as hospital laboratories, both privately owned and public, from a variety of analytical departments responded of which only 21% had no accreditation or certification status (Table 2).

**Table 1:** Number and origin of participants completing the survey

**Country**	**Responders, N (%)**
Albania	16 (1.2)
Austria	67 (5.0)
Belgium	63 (4.7)
Bosnia and Herzegovina	9 (0.7)
Bulgaria	12 (0.9)
Croatia	61 (4.5)
Cyprus	1 (0.1)
Czech Republic	60 (4.5)
Denmark	27 (2.0)
Estonia	8 (0.6)
Finland	21 (1.6)
France	194 (14)
Germany	55 (4.1)
Greece	7 (0.5)
Hungary	16 (1.2)
Ireland	18 (1.3)
Italy	64 (4.8)
Latvia	1 (0.1)
Lithuania	1 (0.1)
Luxembourg	3 (0.2)
Macedonia	21 (1.6)
Montenegro	7 (0.5)
Netherlands	83 (6.2)
Norway	63 (4.7)
Poland	3 (0.2)
Portugal	61 (4.5)
Romania	3 (0.2)
Russia	20 (1.5)
Serbia	54 (4.0)
Slovakia	12 (0.9)
Slovenia	23 (1.7)
Spain	120 (8.9)
Sweden	14 (1.0)
Switzerland	56 (4.2)
Turkey	26 (1.9)
United Kingdom (Great Britain)	75 (5.6)
Ukraine	2 (0.1)
Total	1347 (100)
Answers only by responders who were from EFLM member countries and did NOT state that they do not analyse blood samples.	

**Table 2:** Basic data of participants including the number of laboratories not monitoring preanalytical errors

	**Overall** **(N = 1347)**	**Not monitoring** **preanalytical errors ** **(N = 82)**
	N (%)*	N (%)**
**Please state if you work in a:**		
Primary Care Laboratory	250 (18)	22 (8.8)
Hospital laboratory	532 (38)	36 (6.8)
Laboratory that serves both primary care and hospital (in- and outpatients)	565 (40)	24 (4.2)
**Please state the type of institution you work in**		
Privately owned (for-profit) laboratory	396 (28)	25 (6.3)
Public (non-profit) laboratory	951 (68)	57 (6.0)
**What analytic department do you mainly work in?**		
General Clinical Chemistry	526 (37)	44 (8.4)
I work in many different analytic departments	338 (24)	16 (4.7)
Leading/Supervising position (*e.g*. head of department)	181 (13)	5 (2.8)
Haematology	67 (4.8)	2 (3.0)
Coagulation	13 (0.9)	1 (7.7)
Toxicology/TDM	6 (0.4)	1 (17)
Molecular Biology	12 (0.9)	0 (0)
Microbiology	77 (5.5)	7 (9.1)
Reception/Distribution of samples	8 (0.6)	0 (0)
POCT	8 (0.6)	4 (50)
Quality Management	58 (4.1)	1 (1.7)
Transfusion	5 (0.4)	0 (0)
Clinical Pathology	2 (0.1)	1 (50)
Endocrinology	7 (0.5)	0 (0)
Serology/Virology	2 (0.1)	0 (0)
Other	12 (0.9)	0 (0)
Immunology	22 (1.6)	0 (0)
No answer	3 (0.2)	0 (0)
**Samples *per* day**		
< 500	628 (45)	56 (8.9)
500–3000	505 (36)	21 (4.2)
3001–10,000	177 (13)	5 (2.8)
> 10,000	37 (2.6)	0 (0)
**Is your laboratory accredited, certified or similar? (Multiple answers possible)**		
ISO 15189	593 (44)	11 (1.9)
ISO 17025	68 (5.0)	5 (7.4)
ISO 9001	252 (19)	13 (5.2)
ISO 22870	18 (1.3)	1 (5.6)
National standard	232 (17)	17 (7.3)
Ongoing accreditation / certification	28 (2.1)	2 (7.1)
Other	29 (2.2)	0 (0.0)
No accreditation/certification	289 (21)	44 (15)
Answers only by responders who were from EFLM member countries and did NOT state that they do not analyse blood samples. *Percentage of total. **Percentage of the number of laboratories in the “overall” column. TDM – therapeutic drug monitoring. POCT - point of care testing.		

### Monitoring of preanalytical errors

**Figure 1:**
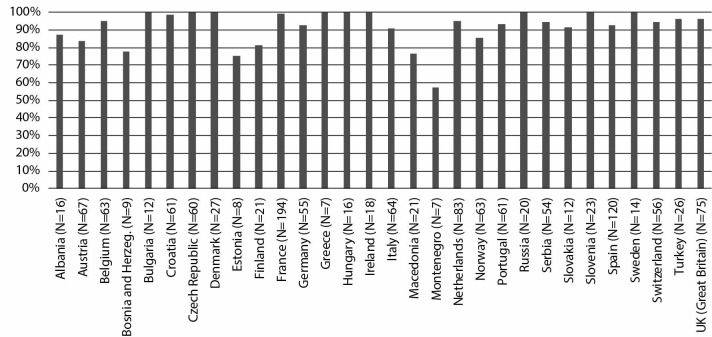
European laboratories monitoring preanalytical errors. The number after the country name in brackets represent the total number of responses from this country. Responses from countries with less than 6 responses are not shown (Cyprus, Latvia, Lithuania, Luxembourg, Poland, Romania, Ukraine).

Of the 1347 participants, 94% (N = 1265) stated they monitored/documented preanalytical errors. These results differed depending on the responder’s home country (Figure 1). Reasons for not monitoring preanalytical errors included the lack of human resources (6%; N = 5), technical issues (*e.g*., lack of specific software) (16%; N = 13), small laboratory/sample size (6%; N = 5), no need and/or only few errors according to the laboratory (7%; N = 6), along with other reasons (20%; N = 16). The answer to this question was left blank by 45% (N = 37) of responders. Nearly half of responders who were not monitoring preanalytical errors (46%; N = 38) declared to be accredited or certified (Table 2, Supplemental table 2). Of those responders who monitored preanalytical errors, 16% (N = 202) used manual documentation of errors (*e.g.* Excel, handwritten, or similar), without these being entered in the Laboratory Information System (LIS). Another 37% (N = 475) entered errors directly into the LIS (either by manual input or automatically) and 43% (N = 543) used a combination of manual and electronic documentation. The remaining 4% (N = 45) of responders did not document preanalytical errors after identifying/measuring them. The results from haemolysis, icterus and lipemia measurement, documentation and usage of the results are shown and discussed in the second manuscript (part 2) of this series (*9*).

### Further processing of preanalytical information

**Figure 2:**
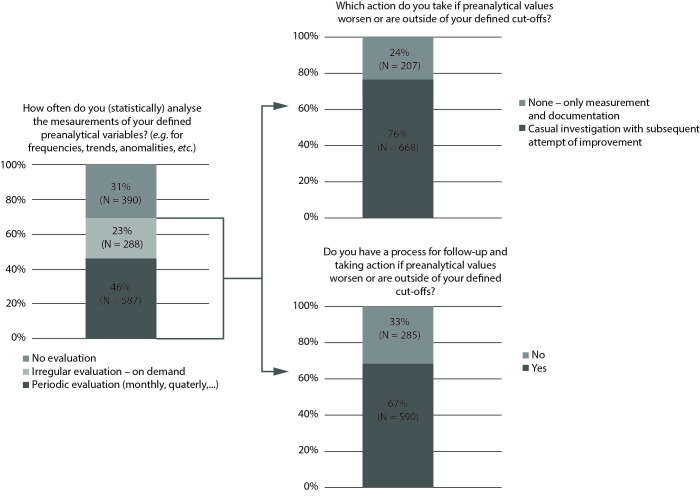
Use of preanalytical data

Of responders actually monitoring preanalytical errors, 31% (N = 390) stated they did not perform further evaluation of these data. Of the remaining 875 laboratories, which did evaluate data from preanalytical errors either periodically or irregularly, 24% (N = 207) stated that this evaluation was not accompanied by improvement actions and 33% (N = 285) of responders claimed that no follow-up processes were initiated when preanalytical values deteriorated or lay outside a defined threshold (Figure 2). Respective accreditation/certification status compared to these responses is shown in Supplemental table 2.

A total percentage of 93% (N = 1255) of responders analysing blood samples stated that they provided preanalytical guidance on laboratory parameters to the clinician/physician/sender either by online-database (34%; N = 465), PDF-/EXCEL-list or a hard copy (10%; N = 141), both online and printed (32%; N = 425) or orally (*e.g*. upon inquiry or within educational training sessions) (17%; N = 224). Of responders not providing information to clinicians (7%; N = 92), 24 stated to be accredited according to the ISO 15189 guideline, which makes up 4% of all respective responders (Supplemental table 2).

### Interest of participants in preanalytical issues and their willingness to actively engage in this topic

When asked about the interest of laboratories in preanalytical topics/issues, 57% (N = 766) of responders stated that they would be interested in participating in an external quality assessment (EQA) program regarding preanalytical errors. Another 14% (N = 185) stated that they were already involved in similar programs. This interest varied between countries. In some, the majority would not be willing to participate, in others, the interest in preanalytical EQA programs was very high. Again in others, such EQA schemes were already in use. A detailed list of the interest in EQA participation sub-analysed by country is included in Supplemental table 3. The interest of laboratories in participating in an EQA program compared to their accreditation/certification status, along with the types of laboratory, is shown in Table 3.

**Table 3:** Interest of European laboratories in preanalytical topics/issues, depending on the type and size as well as on the accreditation/certification status of the laboratory

	**Would you participate in an EQA program regarding preanalytical errors?**			**Would you be interested in an online-platform with current information regarding preanalytical topics (*e.g*. analytical stability of parameters, preanalytical influences on individual parameters, guidelines, *etc*.)?**		**Would you be interested in a guideline for the measurement and evaluation of preanalytical variables?**		**Would you be interested in e-learning programs or webinars on preanalytical monitoring and best-practices?**	
	**Yes,** **N (%)**	**Participating in EQA program, N (%)**	**No,** **N (%)**	**Yes,** **N (%)**	**No,** **N (%)**	**Yes,** **N (%)**	**No,** **N (%)**	**Yes,** **N (%)**	**No,** **N (%)**
**All laboratories** **(N = 1347)**	766 (57)	185 (14)	369 (29)	1177 (87)	170 (13)	1235 (92)	112 (8)	1125 (84)	222 (16)
**Type of laboratory 1**									
**Primary Care Laboratory**	132 (53)	18 (7)	100 (40)	221 (88)	29 (12)	230 (92)	20 (8)	208 (83)	42 (17)
**Hospital laboratory**	299 (56)	61 (11)	172 (32)	453 (85)	79 (15)	481 (90)	51 (10)	436 (82)	96 (18)
**Laboratory that serves both primary care and hospital (in- and outpatients)**	335 (59)	106 (19)	124 (22)	503 (89)	62 (11)	524 (93)	41 (7)	481 (85)	84 (15)
**Type of laboratory 2**									
**Privately owned (for-profit) laboratory**	211 (53)	45 (11)	140 (35)	346 (87)	50 (13)	363 (92)	33 (8)	327 (83)	69 (17)
**Public (non-profit) laboratory**	555 (58)	140 (15)	256 (27)	831 (87)	120 (13)	872 (92)	79 (8)	798 (84)	156 (16)
**Samples per day**									
**< 500**	314 (50)	67 (11)	247 (39)	526 (84)	102 (16)	555 (88)	73 (12)	501 (80)	127 (20)
**500–3000**	313 (62)	75 (15)	117 (23)	452 (90)	53 (10)	476 (94)	29 (6)	437 (87)	68 (13)
**3001–10,000**	114 (64)	39 (22)	24 (14)	164 (93)	13 (7)	167 (94)	10 (6)	156 (88)	21 (12)
**> 10,000**	25 (68)	4 (11)	8 (22)	35 (95)	2 (5)	37 (100)	0 (0)	31 (84)	6 (16)
Accreditation status*									
**ISO 15189**	353 (60)	83 (14)	157 (26)	518 (87)	75 (13)	545 (92)	48 (8)	487 (82)	106 (18)
**ISO 1705**	42 (62)	12 (18)	14 (21)	59 (87)	9 (13)	62 (91)	6 (9)	56 (82)	12 (18)
**ISO 9001**	146 (58)	36 (14)	70 (28)	232 (92)	20 (8)	241 (96)	11 (4)	226 (90)	26 (10)
**ISO 22870**	14 (78)	0 (0)	4 (22)	16 (89)	2 (11)	17 (94)	1 (6)	14 (78)	4 (22)
**National standard**	148 (64)	26 (11)	58 (25)	208 (90)	24 (10)	214 (92)	18 (8)	198 (85)	34 (15)
**Ongoing accreditation**	15 (54)	4 (14)	9 (32)	25 (89)	3 (11)	25 (89)	3 (11)	25 (89)	3 (11)
**Other**	15 (60)	0 (0)	10 (40)	25 (100)	4 (16)	27 (108)	2 (8)	23 (92)	6 (24)
**No accreditation/certification**	145 (50)	40 (14)	104 (36)	239 (83)	50 (17)	256 (89)	33 (11)	234 (81)	55 (19)
EQA – external quality assessment. Answers only by responders from European countries who stated to analyse blood samples. *multiple answers possible. Percentages of subgroups represent the amount within the respective group.									

The majority of responders stated to be interested in a preanalytical online platform (87%; N = 1177), a guideline for the measurement and evaluation of preanalytical variables (92%; N = 1235), and in preanalytical e-learning programs or webinars (84%; N = 1125).

A total of 605 participants responded to the open-ended question “Which preanalytical topics concern you the most?” Results of the qualitative evaluation hereof showed that most text responses were comprehensive and could be categorized without division into meaningful units. The text response generated only three categories: stability of analytes, analytical interference (HIL) and compliance to venous specimen collection guidelines.

Stability of analytes was a concern described as specific analyte stability in samples before and after centrifugation in different test tubes, over time until analysis and the control of transport conditions. Some responders asked for stability tables and others related stability issues to transportation media such as pneumatic tube transport and especially for haemostasis samples. An overwhelming number of responders expressed concerns on analytical interference (*e.g.* HIL) and preanalytical sample handling in order to avoid HIL occurrence as well as EFLM consensus on recommended rejection criteria. The wish for educational programs on how to handle analytical interference also included drugs and autoantibodies. Responders described concerns regarding compliance to venous specimen collection guidelines where traceability of patient identity and samples was an expressed concern as well as various issues on correct sampling practices.

## Discussion

As the preanalytical phase is the part most prone to errors throughout the total testing process, a close inspection and continuous evaluation of this phase is essential to produce high quality test results. Our survey shows that among the 1347 participating European responders analysing blood samples, nearly all (94%) do monitor or document preanalytical errors. These findings are in line with the results of a survey on the use of extra-analytical phase quality indicators (QI) in which Plebani *et al.* found that 90% of surveyed laboratories measured at least one or more extra-analytical QIs/errors (*12*). However, according to our findings there seems to be no standardized way of collecting and documenting these errors, as some responders preferred entering these data directly into their LIS or used a combination of manual and electronic documentation, while others documented these errors manually or not at all after identifying or measuring them. Many/most commercially available LIS lack the preconfigured capability to document quality indicators such as the amount of misidentified samples. Therefore, laboratories are forced to innovate with their own strategies. This is why those responders not monitoring preanalytical errors mentioned a lack of human resources or the LIS to support data collection as the two main reasons hereof. Survey results from Plebani *et al.* were similar to our findings, and led the authors to define the term of the “quality indicator paradox”, a situation with increasing interest in collecting data on extra-analytical errors on one side, but with only few laboratories making regular comprehensive data collections on the other (*13*).

When analysing our findings more closely, there seems to be a clear link between monitoring preanalytical errors and the number of samples processed *per* day by the laboratory. The number of responders not monitoring these errors decreases constantly from 8.9% to 0% with increasing number of samples processed *per* day. This also fits perfectly to some of the reasons these responders gave on why they chose not to focus on preanalytical issues such as “small laboratory/sample size” or “no need and/or only few errors according to the laboratory”. Overall, it seems that responders working in smaller laboratories tend to focus less on topics within the preanalytical phase compared to those employed in bigger facilities. One reason may be that the effort to establish and maintain a rigid quality management system for the extra-analytical phase is similar for either size of laboratories, both financially and in terms of human resources. Another possible reason may be the lack of expertise in preanalytical error handling and documentation in small to very small laboratories.

Another influencing source of variation on whether or not the preanalytical phase is being monitored, is the country of origin of the response, ranging from a 100% to only 57% of responders in countries tracking errors (Figure 1). The reason is unclear but may be influenced by the national society’s activities and local regulatory requirements.

A very interesting finding of this survey was the fact that nearly a third of responders who monitored and documented preanalytical errors, did not evaluate these periodically. Moreover, of those who did, nearly a quarter took no action when preanalytical values worsened or were outside defined ranges. Additionally, nearly half of the responders who monitored preanalytical errors lacked a standardized follow-up process when values worsened or were outside the defined ranges.

Preanalytical errors may be measured and documented by means of reporting them to clinicians alongside the requested test result, thus allowing their accurate interpretation. This does not necessarily mean that these measurements of preanalytical errors are being used for general statistical evaluation. However, only monitoring/documenting these errors without any action taken, based on the individual findings, is undoubtedly not improving the total testing process. Errors like haemolysis rates need to be evaluated not only for specific laboratory reports, but also generally over time on a very detailed level. This enables laboratory professionals to contact the specific health care workers/wards/areas/locations needing attention and to develop ways of improving the situation (*14*, *15*). Tools to approach the problem are freely available when documentation within a LIS is not possible (*16*-*18*).

When providing analytical test results, each laboratory should be concerned about all parts of the total testing process. The role of a medical laboratory is not only to provide test results at a reasonable turnaround time, but also to aid clinicians in choosing the right test, with the right interpretation, and to help medical staff avoiding preanalytical errors in order to maintain high quality analytics (*19*-*21*). To do so, laboratories are encouraged, or even required, to provide information on the assay portfolio including preanalytical instructions to the clinician (*22*). The results of our survey show that nearly all responders across Europe (93%; N = 1255) were following this recommendation in some way. Nevertheless, an online-platform with current information regarding preanalytical topics (*e.g.* analytical stability of parameters, preanalytical influences on individual parameters, guidelines, *etc.*), hosted and kept up-to-date by the EFLM, accessible for everyone, seems reasonable. Laboratories could then refer to this database instead of needing to collect the respective information locally. When asked, nearly 90% of responders would be in favour of such a database. Although different platforms dealing with laboratory parameters are already in place, their major concern is related to their use and interpretation (*23*). As there is currently no platform especially focusing on parameter-specific preanalytical conditions, the EFLM WG-PRE interprets the result of this question as a clear assignment to start building one.

In addition, the interest in a guideline for measurement and evaluation of preanalytical variables was high. Indeed, such guidelines already exist. The International Federation of Clinical Chemistry and Laboratory Medicine (IFCC) Working Group on “Laboratory errors and patient safety” (WG-LEPS) has established a model of quality indicators (MQI), providing instructions on collecting and documenting pre-analytical, analytical and post-analytical QIs, also including the possibility to benchmark local QIs on a national and international level (*24*). Despite continuous efforts in advertising this freely accessible platform, only relatively few laboratories are aware of this possibility (*12*).

To improve knowledge and awareness in preanalytical topics, the EFLM has recently re-launched a completely new and free e-learning platform, offering live webinars and its recordings, along with e-learning courses on many different topics within the laboratory, including pre-analytical issues (*25*). This fits well to the results of the respective question of our survey, where the vast majority of responders stated to be interested in such an educational database.

In contrast, the interest in participating in an EQA program focusing on preanalytical topics was far lower. This finding differed by country. Some countries like Hungary, Croatia, UK, Ireland, Sweden, Norway and Spain had the highest amount of responders already participating in such EQA schemes, which may be linked with national societies or respective EQA providers. The low interest in many other countries might be due to the fact that the portfolios of EQA providers do not include preanalytical schemes. However, the number of respective EQA programs is increasing (*26*-*31*). Similar to the correlation between monitoring preanalytical errors and laboratory sizes, the interest in participating in a preanalytical EQA program was found to be positively correlated with the number of samples processed by respective responders. This confirms our hypothesis that bigger laboratories tend to acknowledge preanalytical issues more than small ones.

Reviewing answers only from laboratories stating to be accredited according to the ISO 15189 guideline, the interest in a preanalytical online-information-platform, in preanalytical guidelines, in e-learning programs or webinars on preanalytical monitoring and best-practices and especially on EQA programs regarding preanalytical errors, were lower than one would expect from accredited laboratories. The ISO 15189 guideline strives to improve quality in laboratory testing by standardizing key processes throughout every laboratory (*22*). Although the demands within this document are quite clear, and some of them exclusively concerned preanalytical processes, it seems that a certain amount of responders claiming to follow this guideline did not adhere to these demands. Although 98% (N = 582) of these responders claim to monitor/document preanalytical errors in general, many of these do not evaluate these measurements, or take any corrective action in terms of a continuous improvement process, and some do not provide preanalytical instructions to clinicians.

At the end of our survey, we asked an open question on which preanalytical issue the responders were most concerned about. We found that most responders were concerned about analytical interference and avoidance HIL as well as recommended rejection criteria. Much has been published on interference of HIL on laboratory tests including a CLSI guideline on interference testing (*32*-*36*). Due to the heterogeneity of manufacturers’ declarations on these interferences, this topic still is a profound concern in many laboratories (*5*, *37*). This is why the WG-PRE has lately called for more transparency in these declarations (*38*). Additionally, the WG-PRE has published recommendations on reporting and managing haemolysed samples (*39*, *40*).

Another major concern of European responders entails data on analyte stability in different samples types and temperatures. This topic is also being addressed by the WG-PRE, partly by providing available data in an easily accessible format and partly by providing consensus on how to retrieve respective data in a standardized way.

A third category of requests from surveyed laboratories regarding compliance to venous specimen collection guidelines has already been fulfilled. The WG-PRE has recently published a joint EFLM-COLABIOCLI (Latin America Confederation of Clinical Biochemistry) recommendation for venous blood sampling which is freely available, and includes extensive additional tools, making it possible for health care facilities to easily standardize the phlebotomy process (*41*).

As limiting factors, we have to mention that although we advised responders to give only one answer *per* laboratory, we cannot rule out that more than one answer was given by the same laboratory. Due to data protection regulations, we refrained from collecting the exact IP addresses of responders. Additionally, we are aware that some countries are overrepresented (*e.g.* France, Spain) while other might be underrepresented. This is at least in part due to differences in the number of laboratories per country. We dealt with this issue by providing country-specific evaluation wherever appropriate.

In conclusion, we show that monitoring the preanalytical phase of the total testing process and acting upon these data varies largely throughout Europe. The interest in preanalytical issues is pleasantly high. With the findings of this survey, the WG-PRE now has confirmation that its members are heading in the right direction with all of the current and planned projects, aiming to harmonize/standardize the preanalytical phase in Europe.

## Supplementary material

Supplementary tables
