# Supplementary material for: European survey on preanalytical sample handling – Part 1: How do European laboratories monitor the preanalytical phase? On behalf of the European Federation of Clinical Chemistry and Laboratory Medicine (EFLM) Working Group for the Preanalytical Phase (WG-PRE)
Source: Biochem Med (Zagreb). 2019 Jun 15;29(2):020704. doi: 10.11613/BM.2019.020704 (PMC6559617; doi:10.11613/BM.2019.020704)
Supplement: Supplementary file 1 — Supplementary tables [file bm-29-2-020704-S1.pdf]

Supplemental material

Supplemental table 1. Survey questions

ID	Questions	Answer options	Only answer this question if the following conditions are met:
**General Information**			
General01	In which country is your laboratory located?	Albania	All
		Austria	
		Belgium	
		Bosnia and Herzegovina	
		Bulgaria	
		Croatia	
		Cyprus	
		Czech Republic	
		Denmark	
		Estonia	
		Finland	
		France	
		Germany	
		Greece	
		Hungary	
		Iceland	
		Ireland	
		Italy	
		Latvia	
		Lithuania	
		Luxembourg	
		Macedonia	
		Montenegro	

		Netherlands	
		Norway	
		Poland	
		Portugal	
		Romania	
		Russia	
		Serbia	
		Slovakia	
		Slovenia	
		Spain	
		Sweden	
		Switzerland	
		Turkey	
		United Kingdom (Great Britain)	
		Ukraine	
		Other	
General02	E-Mail Address		All
	Your email address will only be used in case of any queries regarding your responses to this survey; after evaluation of the survey results, your email will be deleted.		
General03	Please state if you work in a:	Primary Care Laboratory	All
		Hospital laboratory	
		Laboratory that serves both primary care and hospital (in- and outpatients)	
General04	Please state the type of institution you work in	Privately owned (for-profit) laboratory	All
		Public (non-profit) laboratory	
General04b	What analytic department do you mainly work in?	General Clinical Chemistry	All
		Haematology	

		Coagulation	
		Toxicology	
		Molecular Biology	
		Microbiology	
		Reception / Distribution of samples	
		Point of Care Testing (POCT)	
		Quality Management	
		I work in many different analytic departments	
		Leading / Supervising position (e.g. Head of Department)	
		Other	
General06	Samples per day	We don´t analyse blood samples	All
	Please provide the average number of samples processed within your lab on a normal working day.	< 500	
		500 – 3000	
		3001 – 10,000	
		> 10,000	
SQ001	Is your laboratory accredited, certified or similar?	accredited according to ISO 15189	Answer was NOT 'We don´t analyse blood samples' at question '6 [General06]' (Samples per day)
	Check all that apply	accredited according to ISO 17025	
		certified according to ISO 9001	
		certified according to ISO 22870	
		certified according to a national standard	
		No accreditation/certification	
		Other	
**Acquisition of preanalytical variables**			
Aquisition01	Do you monitor/document preanalytical errors?	Yes	Answer was NOT 'We don´t analyse blood samples' at question '6

			[General06]' (Samples per day)
	(meaning errors which happen before the analytical step - e.g. haemolytic, clotted, underfilled samples, ID-errors, etc.)	No	
Aquisition02	How do you monitor/document preanalytical errors?	Manual documentation of errors outside of the laboratory IT-System. (e.g. Excel, handwritten, or similar)	Answer was NOT 'We don´t analyse blood samples' at question '6 [General06]' (Samples per day) and
		Electronic documentation within the laboratory IT-system. (Either by manual input or automatically)	Answer was 'Yes' at question '8 [Aquisition01]' (Do you monitor/document preanalytical errors?)
		Combination of manual and electronic documentation.	
		No documentation (only measurement)	
Aquisition03	Are the exact date and time of blood collection provided with the sample?	Yes, for all samples	Answer was NOT 'We don´t analyse blood samples' at question '6 [General06]' (Samples per day)
		Yes, for most samples	
		Yes, for some samples	
		No (e.g. only the time of order placement is provided)	
		We don´t serve in/out-patients	
		Answers given for In-patients and Out-patients separately (Matrix question)	
Aquisition04	Do you monitor/document haemolysis, lipemia or icterus/jaundice?	Yes	Answer was NOT 'We don´t analyse blood samples' at question '6 [General06]' (Samples per day) and
		No	Answer was 'Yes' at question '8 [Aquisition01]' (Do you monitor/document preanalytical errors?)
Aquisition05	Could you tell us why you don´t monitor/document preanalytical errors?	*text input*	Answer was 'No' at question '8 [Aquisition01]' (Do you monitor/document preanalytical errors?)
SQ001	For which analyses do you monitor haemolysis / lipemia / jaundice?	Haemolysis	Answer was NOT 'We don´t analyse blood samples' at question '6 [General06]' (Samples per day) and
		Icterus / Jaundice	Answer was 'Yes' at question '11 [Aquisition04]' (Do you monitor/document haemolysis, lipemia or icterus/jaundice?)
		Lipemia	

		No H/I/L-check	
		Analyses not performed in my lab	
		Answers given for Clinical Chemistry, Coagulation, Toxicology/TDM and Serology of infectious diseases separately (Matrix question)	
**Haemolysis / Lipemia / Icterus**			
HIL01	How do you measure haemolysis, lipemia or icterus/jaundice?	Automatically by Haemolysis-/Lipemia-/Icterus-Indices.	Answer was NOT 'We don´t analyse blood samples' at question '6 [General06]' (Samples per day) and
	Preferably provide the respective information for your CLINICAL CHEMISTRY analytes. If not applicable, provide information for other analytes (e.g. coagulation, etc.)	Visually by inspecting the sample	Answer was 'Yes' at question '8 [Aquisition01]' (Do you monitor/document preanalytical errors?) and
		Combination of automatic and visual method.	Answer was 'Yes' at question '11 [Aquisition04]' (Do you monitor/document haemolysis, lipemia or icterus/jaundice?)
HIL98	Do you check the quality of your haemolysis- / lipemia- / icterus-measurements by regular internal QC?	Yes	Answer was NOT 'We don´t analyse blood samples' at question '6 [General06]' (Samples per day) and
	Preferably provide the respective information for your CLINICAL CHEMISTRY analytes. If not applicable, provide information for other analytes (e.g. coagulation, etc.)	No	Answer was 'Yes' at question '8 [Aquisition01]' (Do you monitor/document preanalytical errors?) and
			Answer was 'Yes' at question '11 [Aquisition04]' (Do you monitor/document haemolysis, lipemia or icterus/jaundice?) and
			Answer was 'Combination of automatic and visual method.' or 'Automatically by Haemolysis-/Lipemia-/Icterus-Indices.' at question '14 [HIL01]' (How do you measure haemolysis, lipemia or icterus/jaundice?)
HIL04	How do you use information about haemolytic/lipemic/icteric samples in your lab?	The whole sample is rejected	Answer was NOT 'We don´t analyse blood samples' at question '6 [General06]' (Samples per day) and
	Preferably provide the respective information for your CLINICAL CHEMISTRY analytes. If not applicable, provide information for other analytes (e.g. coagulation, etc.)	Only some affected tests are rejected (along with an appropriate comment)	Answer was 'Yes' at question '8 [Aquisition01]' (Do you monitor/document preanalytical errors?) and

		All tests are released with general information on haemolysis/lipemia/icterus	Answer was 'Yes' at question '11 [Aquisition04]' (Do you monitor/document haemolysis, lipemia or icterus/jaundice?)
		All tests are released. No reporting of haemolysis/lipemia/icterus - only documented (e.g. for statistical reasons)	
		Answers given for haemolytic, icteric and lipemic samples separately (Matrix question)	
HIL08	Do you measure triglycerides if the sample had lipemic interference?	Yes	Answer was NOT 'We don´t analyse blood samples' at question '6 [General06]' (Samples per day) and
		No	Answer was 'Yes' at question '8 [Aquisition01]' (Do you monitor/document preanalytical errors?) and
		Sometimes	Answer was 'Yes' at question '11 [Aquisition04]' (Do you monitor/document haemolysis, lipemia or icterus/jaundice?)
HIL09	Do you measure bilirubin if the sample had icteric interference?	Yes	Answer was NOT 'We don´t analyse blood samples' at question '6 [General06]' (Samples per day) and
		No	Answer was 'Yes' at question '8 [Aquisition01]' (Do you monitor/document preanalytical errors?) and
		Sometimes	Answer was 'Yes' at question '11 [Aquisition04]' (Do you monitor/document haemolysis, lipemia or icterus/jaundice?)
HIL10	How do you use cut-offs to define samples as haemolytic?	Parameter specific cut offs as provided by the manufacturer	Answer was NOT 'We don´t analyse blood samples' at question '6 [General06]' (Samples per day) and
	Preferably provide the respective information for your CLINICAL CHEMISTRY analytes. If not applicable, provide information for other analytes (e.g. coagulation, etc.)	In house derived parameter specific cut offs	Answer was 'Yes' at question '8 [Aquisition01]' (Do you monitor/document preanalytical errors?) and
		All samples above a set value regardless of parameter	Answer was 'Yes' at question '11 [Aquisition04]' (Do you monitor/document haemolysis, lipemia or icterus/jaundice?)
		Visual check and individual decision following automated error flag by instrument	
		Visual check and individual decision only	
HIL11	At which general cut-off do you define samples as	0.1 g/L (10 mg/dL) free	Answer was NOT 'We don´t analyse blood samples' at question '6

	haemolytic?	Hemoglobin	[General06]' (Samples per day) and
	(for example: 1 mg/dL of free haemoglobin is equivalent to an Haemolysis Index-value of 1 for Roche Instruments)	0.3 g/L (30 mg/dL) free Haemoglobin	Answer was 'Yes' at question '8 [Aquisition01]' (Do you monitor/document preanalytical errors?) and
		0.5 g/L (50 mg/dL) free Haemoglobin	Answer was 'Yes' at question '11 [Aquisition04]' (Do you monitor/document haemolysis, lipemia or
		1 g/L (100 mg/dL) free Haemoglobin	icterus/jaundice?) and Answer was 'All samples above a set value regardless of parameter' at question '19
		Other	[HIL10]' (How do you use cut-offs to define samples as haemolytic?)
HIL99	Do you use haemolysis measurements to monitor preanalytical quality?	Yes	Answer was NOT 'We don´t analyse blood samples' at question '6 [General06]' (Samples per day) and
	(e.g. haemolysis due to phlebotomy or sample transportation)	No	Answer was 'Yes' at question '8 [Aquisition01]' (Do you monitor/document preanalytical errors?) and
			Answer was 'Yes' at question '11 [Aquisition04]' (Do you monitor/document haemolysis, lipemia or icterus/jaundice?)
HIL97	Which haemolysis cut-off do you use to define low preanalytical quality?	0.1 g/L (10 mg/dL) free Haemoglobin	Answer was NOT 'We don´t analyse blood samples' at question '6 [General06]' (Samples per day) and
	(e.g. haemolysis due to phlebotomy or sample transportation)	0.3 g/L (30 mg/dL) free Haemoglobin	Answer was 'Yes' at question '8 [Aquisition01]' (Do you monitor/document preanalytical errors?) and
	(for example: 1 mg/dL of free haemoglobin is equivalent to an Haemolysis Index-value of 1 for Roche Instruments)	0.5 g/L (50 mg/dL) free Haemoglobin	Answer was 'Yes' at question '11 [Aquisition04]' (Do you monitor/document haemolysis, lipemia or
		1 g/L (100 mg/dL) free Haemoglobin	icterus/jaundice?) and Answer was 'Yes' at question '21 [HIL99]' (Do you use haemolysis measurements to monitor preanalytical quality? (e.g. haemolysis due to phlebotomy or sample transportation))
		Other	
HIL02	Did you verify haemolysis cut-offs declared by the manufacturers?	Yes, all of them	Answer was NOT 'We don´t analyse blood samples' at question '6 [General06]' (Samples per day) and
		Yes, some of them	Answer was 'Yes' at question '8 [Aquisition01]' (Do you monitor/document preanalytical errors?) and
		No	Answer was 'Yes' at question '11 [Aquisition04]' (Do you monitor/document haemolysis, lipemia or

			icterus/jaundice?) and Answer was 'Parameter specific cut offs as provided by the manufacturer' at question '19 [HIL10]' (How do you use cut-offs to define samples as haemolytic?)
HIL96	Please state which protocol you used for verification of serum indices.	CLSI protocol	Answer was 'Yes' at question '8 [Aquisition01]' (Do you monitor/document preanalytical errors?) and
		Local protocol	Answer was 'Yes' at question '11 [Aquisition04]' (Do you monitor/document haemolysis, lipemia or
			icterus/jaundice?) and Answer was 'Yes, all of them' or 'Yes, some of them' at question '23 [HIL02]' (Did you verify haemolysis cut-offs declared by the manufacturers?)
HIL12	Do you use a colour scale for visual haemolysis detection?	Yes	Answer was NOT 'We don´t analyse blood samples' at question '6 [General06]' (Samples per day) and
	Preferably provide the respective information for your CLINICAL CHEMISTRY analytes. If not applicable, provide information for other analytes (e.g. coagulation, etc.)	No	Answer was 'Yes' at question '8 [Aquisition01]' (Do you monitor/document preanalytical errors?) and
			Answer was 'Yes' at question '11 [Aquisition04]' (Do you monitor/document haemolysis, lipemia or icterus/jaundice?) and
			Answer was 'Visual check and individual decision following automated error flag by instrument' or 'Visual check and individual decision only' at question '19 [HIL10]' (How do you use cut-offs to define samples as haemolytic?)
HIL13	In how many categories do you classify haemolysis?	Only "haemolytic" and "Non-haemolytic"	Answer was NOT 'We don´t analyse blood samples' at question '6 [General06]' (Samples per day) and
		3-5	Answer was 'Yes' at question '8 [Aquisition01]' (Do you monitor/document preanalytical errors?) and
		> 5	Answer was 'Yes' at question '11 [Aquisition04]' (Do you monitor/document haemolysis, lipemia or
			icterus/jaundice?) and Answer was 'Visual check and individual decision only' or 'Visual check and individual decision following automated error flag by instrument' at question '19 [HIL10]' (How do you use cut-offs to define samples as haemolytic?)
HIL14	Did you create your sample acceptance policy for samples with lipemia, haemolysis and icteric in a joint collaboration with clinicians?	Yes	Answer was NOT 'We don´t analyse blood samples' at question '6 [General06]' (Samples per day) and

		No	Answer was 'Yes' at question '8 [Aquisition01]' (Do you monitor/document preanalytical errors?) and
			Answer was 'Yes' at question '11 [Aquisition04]' (Do you monitor/document haemolysis, lipemia or icterus/jaundice?)
HIL15	Do you recalculate/correct test results of haemolysed samples using a correction formula?	Yes, for all samples	Answer was NOT 'We don´t analyse blood samples' at question '6 [General06]' (Samples per day) and
	Preferably provide the respective information for your CLINICAL CHEMISTRY analytes. If not applicable, provide information for other analytes (e.g. coagulation, etc.)	Only when requested	Answer was 'Yes' at question '8 [Aquisition01]' (Do you monitor/document preanalytical errors?) and
		No	Answer was 'Yes' at question '11 [Aquisition04]' (Do you monitor/document haemolysis, lipemia or icterus/jaundice?)
**Lipemia**			
LIP01	Do you use sample delipidation for lipemic samples?	Yes - all lipemic samples	Answer was NOT 'We don´t analyse blood samples' at question '6 [General06]' (Samples per day) and
		Yes - but only if potentially affected parameters were ordered	Answer was 'Yes' at question '8 [Aquisition01]' (Do you monitor/document preanalytical errors?) and
		Yes - but only in special situations (individual decision) or in heavily lipemic samples.	Answer was 'Yes' at question '11 [Aquisition04]' (Do you monitor/document haemolysis, lipemia or icterus/jaundice?)
		Yes - but only if requested	
		No	
SQ001	Please state which delipidation method you are using	Centrifugation	Answer was NOT 'We don´t analyse blood samples' at question '6 [General06]' (Samples per day) and
	Check all that apply	Dilution	Answer was 'Yes' at question '8 [Aquisition01]' (Do you monitor/document preanalytical errors?) and
		Spin columns	Answer was 'Yes' at question '11 [Aquisition04]' (Do you monitor/document haemolysis, lipemia or icterus/jaundice?) and
		Refrigeration	Answer was 'Yes - but only in special situations (individual decision) or in heavily lipemic samples.' or 'Yes - but only if potentially affected parameters were ordered' or 'Yes - but only if requested' or 'Yes - all lipemic samples' at question '29 [LIP01]' (Do you use sample delipidation for lipemic samples?)
		Specific reagents (e.g. LipoClear)	
		Other	

**Usage of preanalytical measurements**			
Usage01	How often do you (statistically) analyse the measurements of your defined preanalytical variables?	Periodic evaluation (monthly, quarterly, ...)	Answer was NOT 'We don´t analyse blood samples' at question '6 [General06]' (Samples per day) and
	(e.g. for frequencies, trends, anomalies, etc.)	Irregular evaluation - on demand	Answer was 'Yes' at question '8 [Aquisition01]' (Do you monitor/document preanalytical errors?)
	If the frequency of evaluation differs, depending on the preanalytical variable, please provide the information for the variable which you think is best monitored.	No evaluation	
Usage02	Do you have a process for follow-up and taking actions if preanalytical values worsen or are outside of your defined cut-offs?	Yes	Answer was NOT 'We don´t analyse blood samples' at question '6 [General06]' (Samples per day) and
		No	Answer was 'Yes' at question '8 [Aquisition01]' (Do you monitor/document preanalytical errors?)
Usage03	Which actions do you take if preanalytical values worsen or are outside of your defined cut-offs?	Causal investigation with subsequent attempt of improvement.	Answer was 'Periodic evaluation (monthly, quarterly, ...)' or 'Irregular evaluation - on demand' at question '31 [Usage01]' (How often do you (statistically) analyse the measurements of your defined preanalytical variables? (e.g. for frequencies, trends, anomalies, etc.))
		None - only measurement and documentation.	
**Standardization**			
STD01	How do you provide preanalytical instructions on your laboratory parameters to the clinician/physician/sender?	Online-Database	Answer was NOT 'We don´t analyse blood samples' at question '6 [General06]' (Samples per day)
		PDF/EXCEL-List or hard copy	
		Both online and printed	
		Orally (e.g. upon inquiry or within educational training sessions)	
		We don´t provide preanalytical instructions	

STD02	Would you be interested in an online-platform with current information regarding preanalytical topics?	Yes	Answer was NOT 'We don´t analyse blood samples' at question '6 [General06]' (Samples per day)
	(e.g. analytical stability of parameters, preanalytical influences on individual parameters, guidelines, etc.)	No	
STD03	Would you be interested in a guideline for the measurement and evaluation of preanalytical variables?	Yes	Answer was NOT 'We don´t analyse blood samples' at question '6 [General06]' (Samples per day)
		No	
STD04	Would you participate in an EQA program regarding preanalytical errors?	Yes	Answer was NOT 'We don´t analyse blood samples' at question '6 [General06]' (Samples per day)
	EQA = External Quality Assessment	We are currently participating in such an EQA program	
		No	
STD06	Would you be interested in e-learning programs or webinars on preanalytical monitoring and best practices?	Yes	Answer was NOT 'We don´t analyse blood samples' at question '6 [General06]' (Samples per day)
		No	
STD07	Which preanalytical topics concern you the most?	*text input*	Answer was NOT 'We don´t analyse blood samples' at question '6 [General06]' (Samples per day)

Supplemental table 2. Answers to some of the survey questions filtered by accreditation/certification status

	ISO 15189 (N = 593)		ISO 7025 (N = 68)		ISO 9001 (N = 252)	
	**Yes (%)**	**No (%)**	**Yes (%)**	**No (%)**	**Yes (%)**	**No (%)**
**Monitoring /documentation of preanalytical errors**	98	2	93	7	95	5
**Statistical evaluation of the measurements of defined preanalytical variables? (*e.g.* for frequencies, trends, anomalies, *etc.*)**	74	26	75	25	81	19
**Process for follow-up and taking actions if preanalytical values worsen or are outside of your defined cut-offs**	60	40	65	35	65	35
**Taking action if preanalytical values worsen or are outside of your defined cut-offs**	83	17	91	9	87	13
**Providing preanalytical instructions on laboratory parameters to the clinician/physician/sender**	96	4	97	3	92	8

Supplemental table 3. Interest of European laboratories in preanalytical topics/issues by country

Country	Would you participate in an EQA program regarding preanalytical errors?						Would you be interested in an online-platform with current information regarding preanalytical topics (*e.g.* analytical stability of parameters, preanalytical influences on individual parameters, guidelines, *etc.*)?				Would you be interested in a guideline for the measurement and evaluation of preanalytical variables?				Would you be interested in e-learning programs or webinars on preanalytical monitoring and best practices?				Total
	Yes		We are currently participating in such program		No		Yes		No		Yes		No		Yes		No		
	N	%	N	%	N	%	N	%	N	%	N	%	N	%	N	%	N	%	N
**Albania**	14	88	0	0	2	13	16	100	0	0	16	100	0	0	16	100	0	0	16
**Austria**	18	27	5	7	44	66	48	72	19	28	51	76	16	24	44	66	23	34	67
**Belgium**	51	81	0	0	12	19	60	95	3	5	62	98	1	2	58	92	5	8	63
**Bosnia and Herzegovina**	8	89	0	0	1	11	9	100	0	0	9	100	0	0	9	100	0	0	9
**Bulgaria**	7	58	0	0	5	42	11	92	1	8	12	100	0	0	12	100	0	0	12
**Croatia**	22	36	15	25	24	39	55	90	6	10	59	97	2	3	51	84	10	16	61
**Cyprus**	1	100	0	0	0	0	1	100	0	0	1	100	0	0	1	100	0	0	1
**Czech Republic**	30	50	5	8	25	42	51	85	9	15	54	90	6	10	41	68	19	32	60
**Denmark**	21	78	5	19	1	4	22	81	5	19	25	93	2	7	22	81	5	19	27
**Estonia**	4	50	0	0	4	50	8	100	0	0	8	100	0	0	6	75	2	25	8
**Finland**	10	48	3	14	8	38	15	71	6	29	16	76	5	24	15	71	6	29	21
**France**	119	61	11	6	64	33	175	90	19	10	182	94	12	6	158	81	36	19	194
**Germany**	27	49	3	5	25	45	41	75	14	25	44	80	11	20	35	64	20	36	55
**Greece**	5	71	0	0	2	29	7	100	0	0	7	100	0	0	5	71	2	29	7
**Hungary**	10	63	6	38	0	0	15	94	1	6	15	94	1	6	15	94	1	6	16

**Ireland**	13	72	5	28	0	0	18	100	0	0	18	100	0	0	18	100	0	0	18
**Italy**	35	55	7	11	22	34	57	89	7	11	63	98	1	2	57	89	7	11	64
**Latvia**	0	0	1	100	0	0	1	100	0	0	1	100	0	0	1	100	0	0	1
**Lithuania**	0	0	0	0	1	100	1	100	0	0	1	100	0	0	1	100	0	0	1
**Luxembourg**	3	100	0	0	0	0	3	100	0	0	3	100	0	0	2	67	1	33	3
**Macedonia**	17	81	3	14	1	5	20	95	1	5	20	95	1	5	19	90	2	10	21
**Montenegro**	6	86	0	0	1	14	7	100	0	0	7	100	0	0	7	100	0	0	7
**Netherlands**	49	59	2	2	32	39	59	71	24	29	68	82	15	18	62	75	21	25	83
**Norway**	35	56	17	27	11	17	47	75	16	25	53	84	10	16	53	84	10	16	63
**Poland**	2	67	0	0	1	33	3	100	0	0	3	100	0	0	3	100	0	0	3
**Portugal**	32	52	11	18	18	30	56	92	5	8	59	97	2	3	59	97	2	3	61
**Romania**	2	67	0	0	1	33	3	100	0	0	3	100	0	0	3	100	0	0	3
**Russia**	17	85	1	5	2	10	20	100	0	0	20	100	0	0	20	100	0	0	20
**Serbia**	34	63	3	6	17	31	50	93	4	7	51	94	3	6	52	96	2	4	54
**Slovakia**	6	50	0	0	6	50	9	75	3	25	10	83	2	17	9	75	3	25	12
**Slovenia**	15	65	1	4	7	30	21	91	2	9	21	91	2	9	20	87	3	13	23
**Spain**	71	59	25	21	24	20	116	97	4	3	117	98	3	3	110	92	10	8	120
**Sweden**	8	57	4	29	2	14	12	86	2	14	13	93	1	7	10	71	4	29	14
**Switzerland**	28	50	9	16	19	34	49	88	7	13	49	88	7	13	42	75	14	25	56
**Turkey**	16	62	2	8	8	31	26	100	0	0	25	96	1	4	25	96	1	4	26
**United Kingdom (Great Britain)**	28	37	41	55	6	8	63	84	12	16	67	89	8	11	62	83	13	17	75
**Ukraine**	2	100	0	0	0	0	2	100	0	0	2	100	0	0	2	100	0	0	2
**Total**	**766**	**57**	**185**	**14**	**396**	**29**	**1177**	**87**	**170**	**13**	**1235**	**92**	**112**	**8**	**1125**	**84**	**222**	**16**	**1347**

EQA – external quality assessment. Answers only by responders from European countries who did stated to analyse blood samples (N = 1347).
